# Improving the UNAIDS 90-90-90 Treatment Targets: Solutions Suggested from a Qualitative Study of HIV Patients, Community Advocates, Health Workers and Program Managers in Jimma, Southwest Ethiopia

**DOI:** 10.3390/ijerph17010378

**Published:** 2020-01-06

**Authors:** Hailay Gesesew, Paul Ward, Kifle Woldemichael, Lillian Mwanri

**Affiliations:** 1 Public Health, Flinders University, Adelaide 5042, Australia; hailushepi@gmail.com (H.G.); lillian.mwanri@flinders.edu.au (L.M.); 2 Epidemiology, College of Health Sciences, Mekelle University, Mekelle, Ethiopia; 3 Epidemiology, Institute of Health, Jimma University, Jimma, Ethiopia; kifle.wmichael@gmail.com

**Keywords:** UNAIDS 90-90-90 targets, antiretroviral therapy, delayed HIV diagnosis, discontinuation, HIV care continuum, interventions, immunologic failure, qualitative, Ethiopia

## Abstract

Ethiopia’s performance toward the UNAIDS 90-90-90 targets is low. The present study explored interventions to improve delayed HIV care presentation (first 90), poor retention (second 90) and clinical and immunological failure (third 90). We employed a qualitative approach using in-depth interviews with 10 HIV patients, nine health workers, 11 community advocates and five HIV program managers. Ethical approvals were obtained from Australia and Ethiopia. The following were suggested solutions to improve HIV care and treatment to meet the three 90s: (i) strengthening existing programs including collaboration with religious leaders; (ii) implementing new programs such as self-HIV testing, house-to-house HIV testing, community antiretroviral therapy (ART) distribution and *teach-test-treat-link* strategy; (iii) decentralizing and integrating services such as ART in health post and in private clinics, and integrating HIV care services with mental illness and other non-communicable diseases; and (iv) filling gaps in legislation in issues related with HIV status disclosure and traditional healing practices. In conclusion, the study suggested important solutions for improving delayed HIV care presentation, attrition, and clinical and immunological failure. A program such as the *teach-test-treat-link* strategy was found to be a cross-cutting intervention to enhance the three 90s. We recommend further nationwide research before implementing the interventions.

## 1. Introduction

Human immunodeficiency virus (HIV) is a chronic infectious disease that led to severe epidemics in the last three decades [1]. HIV/AIDS (acquired immunodeficiency syndrome) has been a public health important disease since its emergence three decades ago [1]. According to the 2016 Global Burden of Diseases, 2.4 million people had new HIV infections, 38.8 million people had HIV infection, and 1.2 million people died due to the virus globally [2]. Low- and low-middle- income countries contributed the highest to these figures, including 57% each to the total number of new infections and the total number of people with HIV respectively, and 65% to the total number of deaths [2]. Conversely, high-income countries contributed the least to the above-mentioned global figures, including 2% to the total number of new infections, 4% to the total number of people with HIV, and 3% to the total number of deaths in 2015 [2]. Ethiopia is a low-income country with an HIV prevalence of 0.9% in 2016 [3].

**Figure 1:**
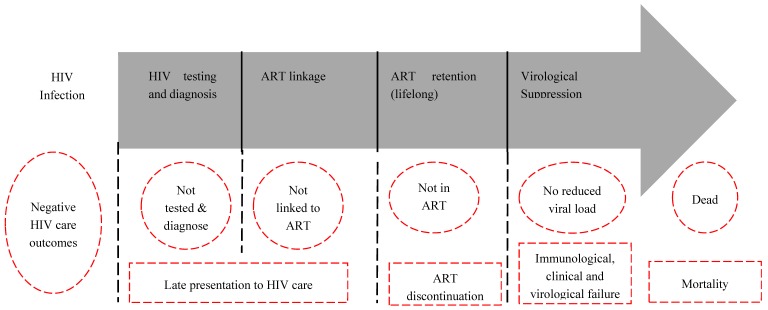
HIV care framework for assessing negative outcomes of HIV care and treatment, Southwest Ethiopia, 2018 (Adapted based on Kranzer et al., 2012) [8]. The box with the grey arrow shows the steps on the pathway of HIV care: HIV testing and diagnosis, ART linkage, lifelong ART retention and virological suppression. The dotted lines, circles or rectangles in red outside the grey arrow show negative outcomes in the pathway of HIV care: late presentation for HIV care, ART discontinuation, immunological, clinical or virological failure, and mortality.

The introduction of antiretroviral therapy (ART) has dramatically reduced HIV transmission and mortality, and improved the survival rate of HIV patients [4]. In Ethiopia, ART program was initially launched in 2003 in twelve hospitals where HIV patients pay for the treatment [5], but the program was provided for free in 22 hospitals since 2005 [6]. In 2015, there were more than half a million (535,069) people with HIV who ‘ever started’ ART in more than 1000 health centres and hospitals [7]. In order to help HIV patients fully benefit from ART and use resources most effectively, Ethiopia uses the HIV care continuum (HCC) framework, initially incepted by the Centres for Disease Control and Prevention in Atlanta in 2013 [8]. The HCC (Figure 1) comprises HIV testing and diagnosis, ART eligibility, lifelong ART retention, and achieving and maintaining viral suppression. Using data from retrospective cohort study (2003–2015) in Southwest Ethiopia, we assessed the whole HCC and found significant proportions of late presentation for HIV care [9], ART discontinuation [10], immunologic failure [11] and mortality [12]—the negative HIV care treatment outcomes described in red in the figure.

To reduce the above-mentioned negative outcomes and further end HIV/AIDS epidemic, the Joint United Nations Program on HIV and AIDS (UNAIDS) and partners proposed three new and ambitious targets [13], commonly called the three 90s. The targets include diagnosing 90% of people living with HIV (first 90), providing ART to 90% of those diagnosed with HIV (second 90), and achieving viral suppression for 90% of patients receiving treatment (third 90). In many developed countries, the performance of the targets has been promising. For example, Sweden was reported to have achieved 90-97-95 [14]. Whereas, the performance of the targets was low in many developing countries, and Ethiopia is no exception. For example, Yemen was reported to have achieved 11% for the HIV diagnosis target and 3% for the treatment target [14]. Of African countries, Botswana achieved a performance that nearly meets the UNAIDS goal—83-87-97 [15]. The 2017 UNAIDS HIV data revealed Ethiopia’s estimated performance for diagnosis, treatment and viral suppression respectively to be 67-59-61 [16]. Similarly, using surrogate measures of our retrospective cohort study, we found the respective performance in Southwest of the nation to be 35-65-66 [17,18]. 

Despite the high magnitude of negative outcomes and low performance of the UNAIDS 90-90-90 treatment targets, the facilitators, barriers and the way forward to improving these outcomes have not been explored very well contextually. Using a socio-ecological model [19], we conducted a qualitative study to understand facilitators, barriers and interventions to improve the negative outcomes of HIV care and treatment via interviewing HIV patients, health care workers, community representatives and program managers from Southwest Ethiopia [20]. In brief, the barriers included HIV stigma, local traditions (e.g., traditional healing and patriarchal society), free ART as expensive (e.g., selling ART in black markets), poor availability and accessibility of HIV care services, and fragmented health care system. Details of the socio-ecological model and barriers and facilitators of HIV care and treatment were published elsewhere [20]. The focus of this paper is to explore interventions to address the above barriers to improve the entire HCC.

## 2. Materials and Methods

### 2.1. Study Design, Setting and Participants

A qualitative study was applied to explore possible ways to improve HIV care and treatment. Conducted between October 2017 and February 2018, we employed one-on-one face-to-face in-depth interviews among the following study participants from Jimma, Southwest Ethiopia:

(a) HIV patients visiting Jimma University Teaching Hospital (JUTH) and Jimma Health center,
(b) HIV-care providers working in ART clinic in JUTH and Jimma Health center,
(c) Communities advocates from HIV patients’ association, religious groups, ‘*Idir*’ (local association at the community level), women association and health extension workers (HEWs), and
(d) HIV-care system administrators working at governmental and non-governmental organizations.

### 2.2. Participant Recruitment Procedure

The patients were purposively recruited after receiving the service from the clinic. The ART nurse or physician invited the potentially eligible patients to the study through provision of a letter if they were willing to be a potential study participant for research on HIV care and linked to the interviewer. The interviewer introduced himself through a letter of introduction, provided the study information through an information sheet, and asked to sign a consent form. The letters were translated into the local languages. Similarly, the HIV care providers, community representatives and HIV care system administrators were also recruited purposely. We have considered a mix of gender, rurality and history of lost-to-follow-up or retention as purposive criteria to recruit patients with HIV. In addition, gender, professional and stakeholders mix were the purposive criteria to recruit the HIV care providers, community representatives and HIV care system administrators. The aim of including a different mix of study participants was to get saturated information on the issue.

### 2.3. Data Sources, Instruments and Data Collection Processes

The primary data were obtained by interviewing HIV patients, HIV-care providers, advocates or community representatives, and HIV-care administrators using a prepared in-depth interview guide. The guide comprises the following constructs: ways that improve individual-level factors (knowledge, experience, expectations, attitudes, beliefs and disclosure), community-related factors (care and support, stigma and discrimination, and traditional healing), healthcare provider-related factors (quality of care, interaction with HIV health care providers, referral and linkage, logistics, availability, administration and capacity building), and policy-related factors (health policy, HIV/AIDS policy, health care financing, guidelines and standards, new programs, and integration with NGOs). Pilot interviews were conducted in order to test the interview guide and the translation or transcription process in order to accommodate necessary modifications. The actual interview took from 38–120 min for each participant and this was clearly conveyed in the letter of introduction and information sheet. All participants were interviewed in a quiet, secured and confidential area. 

### 2.4. Data Management and Analyses

Audio-recorded semi-structured interviews were carried out, and hand-written field notes were taken immediately. Each interview was transcribed word by word. The following notations were used within the transcripts: (1) (*health facility*)—Parenthesized words are possible hearings; (2) *NO NO*—Capitals, except at the beginning of lines or as acronyms, indicate loud sound relative to the surrounding talk; (3) *Yeah* (.2)—Numbers in parentheses showed elapsed time in silence tenths of seconds; (4) *You know what they do*?—Punctuation indicates speaker’s intonation; and (5) ‘*Tsebel*’—Words under single quotation are locals. 

Data analyses were employed along with data collection. Shortly after conducting each interview, the researcher read the full verbatim transcript several times. The resulting data were analysed according to the thematic framework analysis approach for qualitative data analysis [21,22,23] using NVivo for Mac 11.4.1 (QSR International Ptd Ltd., Doncaster, Victoria, Australia) [24]. Thematic framework analyses approach included data familiarization, reading and re-reading the transcripts, generating initial codes, developing a working analytical framework and grouping codes into themes. The analyses were both deductive and inductive type so that categories known *a priori* and emerged from the data were established. The themes and codes were presented and interpreted. In addition, we included illustrative quotes to support the themes. While presenting the illustrative quotes, we used (i) ‘*PHo*’ and ‘*PHC*’ to indicate quotes from patient participant from hospital and health centre respectively; (ii) ‘*HWHo*’ to indicate a quote from health worker participant from a hospital; (iii) ‘C’ to indicate a quote from community advocates or representatives; and (iv) ‘*Admin*’ to indicate a quote from a participant from HIV program manager group. Trustworthiness—credibility, transferability, dependability and reliability—of data was also ensured using several techniques [20,25,26].

### 2.5. Ethical Approval

Ethical approval was obtained from the Social and Behavioural Research Ethics Committee of the Flinders University (Project No: 7698) and the Institutional Review Board of Jimma University (Ref. No: IHRPG/878/2017). Permission from the respective institutions was taken to approach the study participants. Written consent was sought prior to the interview. All study participants were assured that the information they gave would be treated with the strictest confidence and no identifying information would be published. It would remain confidential and would not be shared with any party without their knowledge or consent. However, complete anonymity could not be guaranteed given the involvement of the ART nurse or physician.

## 3. Results and Discussion

### 3.1. Demographic Characteristics of Study Participants

A total of 35 participants were interviewed, and these represented a variety of organizations and positions throughout Jimma including Jimma Zone Health Department, Jimma HIV/AIDS prevention and control office (HAPCO) and Jimma Town Health Office, and non-governmental organizations such as Organization Service for Social Aid (OSSA), Family Guidance Association (FGA) Confidential, Marie Stopes International, and the International Centre for AIDS Care and Treatment Program (ICAP) in the Southwest regions of Ethiopia. Table 1 summarizes the demographic characteristics of all respondents included in the in-depth interviews. 

### 3.2. Key Solutions to Improve HIV Care and Treatment

Four key themes emerged as solutions for HIV care and treatment from the analysis. These included strengthening existing programs, implementing new programs, decentralizing and integrating services, and filling gaps in legislation. 

#### 3.2.1. Theme 1: Strengthening Existing Programs

The following codes were listed under this theme: strengthening previous programs, involving religious groups, and using ‘*toko-shenni*’ (‘*toko-shenni*’ is a local term to mean ‘one-to-five’ networks, denoting five-member households and a convenor, and is organization of households by their neighbourhood to execute health, and other social activities relevant to the community.) or one-to-five networks. It is recognized that the government of Ethiopia has adapted and designed a number of relevant programs for comprehensive HIV/AIDS prevention, treatment, care, and support services. Programs including community awareness, voluntary counselling, testing campaigns and others have been initiated in Ethiopia [27,28], but are currently insufficiently performing. The current study participants, with one voice, mentioned a lack of consistent commitment to implementing these programs as part of reasons for the underperformance of these programs (see Box 1, Quote 1), as also supported by other studies [29,30].

Box 1 **Examples of interventions: strengthening exiting programs.**

*What I suggest is the government should work hard like at the beginning. Every activity was taken seriously during HIV emergence—the awareness creation, the VCT (voluntary counselling and testing) campaign, the involvement of NGOs,* etc. *Such courage should comeback… The commitment of every individual should be as it used to be. (HWHo—07)*
*…the religious leaders themselves should do tell that patients can take their medication while they are visiting the religious places. Patients listen more to their religious leaders than the health workers. (PHC—03)*

*I would suggest integrating HIV testing program into the “toko-shenni” or (one-to-five network). Government is using this program for its politics, why not for health then ha … (Laughing)? (PHo—08)*

The quote also shows the need to reinvigorate the existing programs and to address the perceived negligence which seemed to exist at each level represented by the study participants—community, health workers and the program managers. 

The participants also acknowledged the need to involve and/or link with religious leaders, but the support and regular follow-up were inadequate. Partnerships development with religious leaders seemed to be a necessity to reduce late ART initiation and attrition. Because a number of patients failed to commence or withdrew from ART when visiting traditional or religious healing places. Quote 2 in Box 1 demonstrates the importance of religious leaders in counselling patients to use modern and traditional healing simultaneously. The collaboration with religious leaders could begin by actions as simple as considering HIV counselling within their congregation. Nowadays, there are some promising activities involving religious leaders in HCC programs. For example, Management Sciences for Health (MSH) [31] is piloting a program called Ethiopia Network for HIV/AIDS Treatment, Care and Support (ENHAT-CS), a pilot project in Tigray and Amhara in North Ethiopia [32]. The program supports patients with HIV from selected health centres and hospitals who visit traditional healing places, and it is conducted in partnership with Ethiopian HIV positive people and religious associations. These activities help patients to know their HIV status and link to HIV care, a goal of the first 90 of the UNAIDS targets.

Structures available for other purposes such as the new model or network called “one to five, locally called *toko-shenni*” [33] can also be used to run HIV care programs in Ethiopia. The convener in the *toko-shenni* organizes activities and facilitates meetings to discuss various issues such as health, agriculture, education, security and other as packages relevant to the community. Furthermore, the leader mobilizes, monitors, and trains five others or five households on the issues of interest or packages. The participants in this study suggested that HIV counselling and testing could be included as one of the packages in this network (see Box 1, Quote 3). Even though there are no studies supporting the use of one-to-five networks in HIV care, the networks have been used to improve immunization coverage in pastoral communities in Ethiopia [34], indicating that this avenue could be scaled up for use in the HIV care program.

#### 3.2.2. Theme 2: Implementing New Programs

In addition to strengthening existing programs, it is also necessary to implement new programs to meet the ambitious UNAIDS 90-90-90 treatment targets. The following new programs were suggested by the study participants: self-HIV testing, house-to-house HIV testing, community ART distribution, and *teach-test-treat-link* strategy. 

Self-HIV testing (SHT) is a screening process whereby, privately, a person collects a specimen, performs a test, interprets the result and refers themselves to a health worker for confirmation of any positive result. This program was supported by more than half of the study participants. They mentioned advantages like preventing HIV transmission to others, linking to ART care early and reducing stigma (see Box 2, Quote 1). The above quote suggests that SHT could be significant to individuals through early self-detection of HIV status and timely HIV care seeking so as to meet the HIV diagnosis targets.

Box 2 **Examples of interventions: implementing new programs.**

*… village merchants have the money but they are not educated and they can do whatever they like. If they ask a village girl for sex, none will say ‘no’. In addition, those merchants can officially have 4 or 5 wives … Imagine what will happen if he got infected by HIV. So, if they are able to test themselves, at least, they know their status and they may not infect others. And if it is for free, this will be outstanding. It will also help to reduce the fear of stigma. (PHC—04)*

*House to house HIV testing? NO NO, people (who are diagnosed through House-to-House HIV testing) may not come to care or be compliant after linkage. If the testing is especially conducted by HEWs, there may be fear of stigma. By the way, HEWs lack motivation and responsibility. (Admin—05)*

*Yes, we are doing this (CAGs) and it doesn’t have a problem. We have three cases who come from a very far place and they don’t want to take from the nearby clinics for the fear of isolation. So, they collect their pills in rotation. Similarly, husband takes his wife’s medication and wife takes her husband’s. But now because we have the ‘appointment spacing model’, I don’t think we need it. (HWHC—05)*

*Peer educators are HIV+ people and it is easy for them to share their story and experiences, so that other HIV+ people easily accept their lived experiences. When they give a witness how ART helps to be what they are now, people start to lose their fear of HIV testing or going to health facilities to get HIV treatment … so, yeah, this will be an outstanding. You see, the benefit is also for them (peer educator) because they are getting a little monthly salary… (C—03)*

Additionally, this program would also be significant to the general public through a reduction in HIV transmission, as is well known that HIV treatment is prevention [35]. The quote also indicates that SHT may be effective for special groups in the population who are at risk of HIV, who need expensive regular testing. Because their needs would be met due to the availability of free HIV testing kits through the SHT program. Conversely, some participants opposed the implementation of this new program citing several reasons including a fear of failing to cope with emotions (e.g., shock or committing suicide), fear of failing to link with care, revenge, shortage of testing material, and fear of insufficient technique when testing, which could lead to incorrect or false results with severe disadvantage. Despite these, some African countries such as Kenya [36], Malawi [37] and Zimbabwe [38] are implementing the program with positive outcomes. For example, in Kenya, the rate of HIV positive was found higher among people on standard HIV testing and SHT than on standard HIV testing alone [39]. 

House-to-house (H2H) testing was also another intervention to improve the first 90 and was suggested by more than half the participants. H2H is an approach whereby HIV testing is undertaken in every house by peer educators, HEWs or trained lay counsellors. Supporters of this program noted the following advantages: reducing stigma, availability of services particularly for those patients who may be bedridden at home, preventing HIV transmission to others, and timely linkages to ART care. Particularly, participants emphasized that this program has a considerable impact in reducing HIV related stigma. On the other hand, other participants raised the following challenges: difficulties in disclosure, increase in family stigma, fear of confidentiality, failing to cope with emotions and a potential shortage of HIV kits due to the system being overwhelmed by requests (see Box 2, Quote 2). Interpreting the quote, H2H may not be successful if HEWs are involved in the screening program. This may be because most HEWs are drawn from their home locations and people fear lack of confidentiality regarding their HIV status. This assertion could also be an indication of limited trust in these community health workers by members of the communities they serve. However, H2H has been implemented and found to be effective in some African countries, including Kenya, Malawi and Zimbabwe [40,41].

Community ART groups (CAGs) was another new solution to meet the second 90 as suggested by some participants. CAG is a program in which people who are HIV positive and who disclose their HIV status to other patients nearby form a group and collect pills for the group in rotation or via their representatives. Like other new suggested programs, CAG was supported by some study participants and opposed by others (see Box 2, Quote 3). Even participants who supported the Appointment Spacing Model—a program that enables patients to receive a six months ART prescription—thought that CAGs was also an important program. Participants who were in favour of this program identified the following benefits: stigma can be reduced, group members can establish their own community, and patients can save their energy, time and transportation costs particularly those from remote areas. On the other hand, participants who were against this program argued that drugs may be misused (lost, sold and exchanged), people may not be compliant so that there could be associated complications such as the development of treatment failure, and HIV status disclosure may be a challenge. This program has been successfully implemented in Mozambique, Malawi, South Africa and the Democratic Republic of the Congo [40].

The *teach-test-link-trace* (TTLT) model through peer educators and HEWs, was also another new program which emerged from the analyses, a crosscutting intervention for the three 90s. Peer educators are trained patients with HIV who have disclosed their HIV status publicly. Study participants acknowledged that these peer educators encouraged other patients with HIV to link into and remain in HIV care. The peer educators convince most patients with HIV who are resistant to starting ART when counselled by health workers, and also trace patients who have been lost from HIV care. It is also evident that the HEWs in Ethiopia visit every house on a daily basis to implement their 16 packages in four groups including disease prevention, family health, hygiene and environmental sanitation, and health education and promotion [42,43]. Therefore, in cooperation with HEWs, it is possible to assign peer educators to counsel people about HIV (*teach*), perform HIV testing (*test*), link HIV positive patients to HIV care (*link*) and trace lost patients house-to-house (*trace*). A new term called the TTLT model was coined for the approach (see Box 2, Quote 4). The importance of peer educators in the HCC was reported in several studies [40,44,45,46,47].

#### 3.2.3. Theme 3: Decentralizing and Integrating Service

History has provided a witness to the success of decentralization and task shifting in the context of ART services in Ethiopia [27], and other countries [48,49]. Initially, ART in Ethiopia was provided in selected public hospitals, and it has now been decentralized to the level of health centres [27]. Similarly, ART was once prescribed only by specialized internists, but currently, a diploma-level nurse can prescribe it. More recently, the involvement of peer educators has had a dramatic impact in reducing ART discontinuation. Participants in the present study suggested decentralizing services to private clinics and health posts (a kebele, lowest administrative unit in Ethiopia, based health service centre that serves for 3000–5000 people), and shifting the task to HEWs. Decentralization of ART to the level of health centres or district level hospitals has been promoted by other scholars from Africa [48,49]. 

The participants in the current study noted the substantial impact of HEWs in reducing maternal and child health morbidity and mortality, and they predicted similar achievements in HIV. Thus, they recommended the expansion of services into every community by providing ART in health posts (see Box 3, Quote 1). On the other hand, there were participants who questioned the success of decentralization and task shifting. According to them, it was suggested that HEWs were overloaded, had less capacity and commitment to manage HIV, and were not trusted to keeping confidentiality by the community (see Box 3, Quote 2). A HEW who oversees a health post also supported that it is not feasible to provide ART in health posts due to reasons such as insufficient training and poorly resourced workplaces (see Box 3, Quote 3). From the above quotes, the decentralizing of ART care services was challenging from the perspectives of both the patients and the HEWs. In addition to the opposition of the task shifting by the HEWs in charge of health posts, trust in the HEWs was also described as a challenge by other study participants. There are countries, however, who use community health workers to distribute ART. In these countries, the community health workers are only responsible for ART distribution, not other health activities. In Ethiopia, HEWs are responsible for implementing the 16 packages and it could be a significant challenge for them to provide ART care services as well [50,51].

Box 3 **Examples of interventions: decentralizing and integrating service.**

*… the service (ART care services) should go to the community or to the people out there. For sure, it will be started in the health post. It will not be a problem too if ART is started in the private clinic but the government should support a budget since they are organizations for profit. History tells that it is possible. Initially, ART was prescribed by specialists and then by general practitioners in hospital, then by health officers and nurses in health centre. So why not by HEWs in the health post? (HWHo—06)*

*Let me tell you one case. One patient defaulted from ART care, and the nurses gave to trace the patient’s address to a HEW. She (the HEW) met him (defaulted patient) in a market and she said … “Hey, why don’t you attend your ART clinic?”. This is in a market where mass is gathered, and you can imagine how he feels ashamed and traumatized. Thus, there are people who don’t accept and trust those HEWs. (C—01)*

*ART in health post? Well, with this issue, one, we need to have a training. Second, 80% of our work is field work—we are in office only on Friday, once a week. So, people may not see us and default or likes may happen. The office arrangement does also not allow for ART drugs to store in a way it should be. Yeah … it needs a lot of resources. And the other is we are too busy—we are collecting taxes, organize microfinance, doing other non-health related activities. So, we couldn’t provide ART in health post. (C—03)*

*ART in private clinics is good. Because, there are rich people who don’t need to see public clinics so we can get those patients through private clinics. By the way, we are having a plan to start ART in one private clinic. I have told you that we start HIV testing in selective private clinics, and we propose three private clinics for this year. (Admin—01)*

With regard to ART in private clinics, some study participants opposed launching ART services in private clinics citing reasons including lack of the capacity to run programs and potential misuse of the drugs. On the other hand, study participants who had observed the successful management of tuberculosis program in private clinics proposed the commencement of ART services in the private clinics (see Box 3, Quote 4). Furthermore, ART in private clinics has been achieved in private hospitals in Addis Ababa, the capital city of Ethiopia [48]. Alternative suggestions have been made including the potential to introduce a public–private partnership in which health workers from private clinics would treat patients in public hospitals or health centres [52]. 

#### 3.2.4. Theme 4: Filling Gaps in Legislation

The legislation of the Federal Democratic Republic of Ethiopia (FDRE) has laws related to HIV. The revised criminal code (2005) provides for punishment up to and including the death penalty for the intentional spread of HIV. The legislation also guarantees the right to privacy (confidentiality) of HIV status except for children, bedridden or seriously ill patients, and mentally incapable patients. Yet, there are gaps within the existing legislation. Participants in the study identified gaps in laws about disclosure and traditional healing and stated that these gaps were challenges to achieving the desired outcomes of ART. For example, the gap in disclosing partner’s HIV status is one of the sensitive issues. A husband who does not disclose his HIV status to his pregnant wife prevents her and the baby receiving HIV care services, affecting benefits from early HIV diagnosis and ART initiation, and subsequently the performance of the three 90s. Similar problems occur if a mother who is HIV positive does not disclose her status to her husband. Thus, participants in the present study suggested the need to develop legal provisions to mandate notification of HIV status to one’s partner (see Box 4, Quote 1).

Box 4 **Examples of interventions: filling gaps in legislation.**

*If a husband for instance doesn’t disclose his HIV status for his wife and if his wife got pregnancy, this is a crime and is not different from stabbing to death. He is infecting two lives at the same time. This is happening. We have to try our endeavor to counsel them to disclose their status and bring their partners to care. If this fails, the partner should be sued. I can’t see this is clashing with confidentiality. They are husband and wife and this should be treated differently. (C—04)*

*As a pastor, I have told you earlier that my job is to pray for people, not to order to discard their pills. Recently, three people with HIV came to me to cure them from the virus. Then, I had to pray, pray and pray in front of God (.2), and finally three of them cured by my service. They went to re-check and were negative. I am right to declare the cure (HIV) but I shouldn’t order to throw pills. However, there are pastors who emotionally order people to discard pills, and they should be asked by law—they do this to get acceptance from the attendants. I support considering this to be included in the legislation. (C—10)*

The quote reflects that partners need to disclose their HIV status to each other for the sakes of themselves and their child/ren. There are inconsistencies in laws in countries and among states within a country with regard to mandatory disclosure of HIV status to a partner. In Australia, HIV positive people in Tasmania are required to disclose their status before sexual contact, and similarly before sexual intercourse in New South Wales, but this is not required in Victoria [53]. In the United States, the penalty for nondisclosure in North Carolina is stricter than in Alabama [54].

Another issue related to gaps in legislation covering HIV care provision is the importance of implementing a legal framework regarding traditional healers. The patients noted that there are traditional health practitioners who declare an HIV cure and obligate patients with HIV to stop their treatment. A pastor declaring that a person with HIV is cured and telling that person to discard the pills can be accused of killing or attempting to kill that person. Furthermore, the pastor is providing false witness if this person is not cured. Similar activities happen in the Orthodox religion. These scenarios are against the law in some countries, and yet they are the most difficult to overcome (Box 4, Quote 2). These quotes demonstrate the role of traditional healers in modern HIV care management. Examples such as Orthodox priests not allowing patients to bring pills to Holy Water places, Protestant Christian pastors broadcasting on live TV shows that they have cured HIV by praying, and religious fathers passing a rule for followers to discard pills led participants to consider the need for legal provision. The participants noted that the negative consequence of the absence of these legislations was higher among women than men, as women were more likely to visit traditional healers and be affected by stigma than men. Nevertheless, they pointed out the problems of filling these gaps systematically. In South Africa, it is against the law for traditional healers to treat undiagnosed patients with HIV, and they are obliged to refer people to a hospital if they visit their place before diagnosis [55]. 

Previous studies have revealed that interventions that improve early HIV diagnosis and linkage to HIV care and strategies that enhance retention improve the goal of virological suppression [14,56,57,58]. For example, a clinical trial in Uganda revealed that patients with HIV who had peer support had higher virological suppression rates than those who had not [57]. Similarly, the *teach-test-link-trace* strategy, a peer support provider-based program, suggested from our study will improve the third 90. Another randomized clinical trial in Uganda also depicted that home-based ART care improved immunological success [58]. Similarly, the CAGs program suggested from this qualitative study could also improve ART adherence and subsequently clinical and immunological success. Hence, the interventions mentioned to improve the first and second 90s will improve the third 90.

Overall, the entire discussion about improving HIV care and treatment in this paper demonstrated the intertwined effect of various ecologic levels on individual HIV care practices but in doing so, it interplayed the effects of other SEM levels. We will demonstrate the relationship between gender and negative HIV care outcomes as an example to show these interlinked effects. The study revealed that women were found to have a higher risk of negative HIV care and treatment outcomes than men. Because they made fewer visits to HIV clinics and failed to access the care, a concept that shows the effect at the microsystem level. The study also found that stigma related to HIV, traditional healing practices and living in a male-dominated society contributed to women’s poor access to HIV care, demonstrating issues operating at mesosystem level. Furthermore, the study depicted that there was a lack of policies to empower women, address stigma related to HIV at multiple ecologic levels, implementing an effective collaboration between modern and traditional health practices. Also, there were gaps related to legal issues about the disclosure of HIV status to partner and malpractices by traditional health practitioners about HIV treatment. These demonstrate the concept of SEM at the macrosystem level. We can apply similar interpretations to other characteristics of HIV patients in this study. 

The study has the following limitations. First, some argue that the choice of male interviewer over female interviewee may affect the data collection process. Nevertheless, the researcher (male) was the interviewer who knows the local language, norm and concerns raised by women. Furthermore, some participants may also have the willingness to discuss some issues given the interviewer is an educated professional. Second, the number of HIV care program managers was small (n = 5); however, the total number of study participants were (n = 35) was within the acceptable range for qualitative studies. In addition, most findings from the four groups complement each other. Third, we did not include participants from private health facilities and we might miss opinions of patients attending private HIV clinics, HIV care providers working in private HIV clinics and community representatives supporting HIV patients in private ART clinics on the issue. Nevertheless, the study participants involved in the study discussed their opinions on HIV care and treatment issues in private clinics. Lastly, the study participants were only from Jimma, and females were the majority of the study participants questioning whether more contextual factors were explored. However, we reached data saturation. 

## 4. Conclusions

The study recommends that early diagnosis could be improved through self- and home-based HIV testing, ART linkage and retention through community ART groups, integration and collaboration with religious leaders. Additionally, disclosing one’s HIV status seems to be a key to obtaining HIV care support, therefore, the need for promotion of disclosure including mandatory notification to a partner was suggested. The *teach*, *test*, *link and trace* model was found to be one of the effective interventions that improves the entire HIV care continuum. We, however, recommend further advanced research (e.g., experimental studies) to assess the acceptability rate and effectiveness of the suggested new programs in a wider context to explore the drawbacks of each intervention and shed light on further strategies to end the HIV/AIDS epidemic. 

## Figures and Tables

**Table 1:** Demographic characteristics of respondents.

Characteristics, N = 35		Patients with HIV, N = 11 (31.4%)	HIV Care Providers, N = 9 (25.7%)	Community Advocates, N = 10 (28.6%)	HIV Care Administrator, N = 5 (14.3%)
Age	Median (range), years	33 (21–42)	40 (27–50)	32 (28–48)	35 (27–42)
Sex	Male	2	4	3	3
	Female	9	5	7	2
Religion	Orthodox	8	5	6	3
	Muslim	1	1	3	2
	Protestant	2	3	1	0
Ethnicity	Oromo	2	5	9	2
	Amhara	4	3	1	3
	Debub ^†^	5	1	0	0
Education	Not read and write	1	---	0	---
	Primary	4	---	1	---
	Secondary	4	---	2	---
	College and above	2	9	7	5
Marital status	Never married	2	---	2	1
	Married	5	---	8	4
	Separated or divorced	4	---	0	0
Residence	Urban	7	---	5	5
	Rural	4	---	5	0
ART Status	Had history of LTFU	5	---	---	---
	Retained since on ART	6	---	---	---
Position	HIV volunteer	---	---	3	---
	Religious leaders	---	---	3	---
	Coordinator or expert	---	7 ^^^	4 ^&^	2 ^$^
	Program manager	---	2	---	3
Total work experience *	Median (range), years	---	20 (6–32)	8 (3–28)	19 (7–26)
Work experience ^#^	Median (range), years	---	6 (2–12)	8 (3–12)	3 (2–12)
Time since on ART	Median (range), years	9 (2–16)	---	---	---
Average time to reach ART clinic	Median (range), minutes	30 (5–240) by transportation or 27(15–45) on foot	---	---	---

^†^ Debub refers to people who come from Gguragie, Wollayta and Keffa ethnic groups—refers to *Not Applicable or No information*; ^^^ All were ART nurses from hospital or health centre; ^&^ Two were urban health extension professionals and two were rural health extension workers from health posts; ^$^ Both were coordinators and counsellors from non-governmental organizations; * Total work experience in career; ^#^ Work experience in the current institution; LTFU: lost-to-follow-up.
